# Iranian midwives’ attitudes and beliefs toward physiological childbirth: a cross-sectional study

**DOI:** 10.1186/s12884-019-2509-y

**Published:** 2019-10-12

**Authors:** Narges Sadeghzadeh, Leila Amiri-Farahani, Shima Haghani, Syedeh Batool Hasanpoor-Azghady

**Affiliations:** 1 Department of Reproductive Health and Midwifery, School of Nursing and Midwifery, Iran University of Medical Sciences, Tehran, Iran; 2 Department of Reproductive Health and Midwifery, Nursing Care Research Center (NCRC), School of Nursing and Midwifery, Iran University of Medical Sciences, Tehran, Postal code: 1996713883 Iran; 3 Nursing Care Research Center, Iran University of Medical Sciences, Tehran, Iran

**Keywords:** Attitude, Belief, Midwives, Medicalization of childbirth, Physiological childbirth

## Abstract

**Background:**

The significant role of midwives in providing labor and delivery care underscores the necessity of the identification of attitudes and beliefs of these groups of maternity care providers toward physiological childbirth. The purpose of the current study was to describe midwives’ attitudes and beliefs toward physiological childbirth and identify its related factors.

**Methods:**

This cross-sectional study was carried out on 200 midwives working in the labor and delivery wards of selected hospitals in an urban area of Tehran, Iran, through the continuous sampling method from May to July 2018. The data were collected using a two-part demographic characteristic form and Midwives’ Attitudes and Beliefs Questionnaire-Revised toward physiological childbirth. Data analysis was performed in SPSS software (version 25). The significance level was set at *P* < 0.05.

**Results:**

The mean score of midwives’ attitudes and beliefs toward physiological childbirth were 119.90 with a standard deviation of 9.30. Moreover, of the different domains of Midwives’ Attitudes and Beliefs Questionnaire-Revised, the women’s experience of birth (78.53) and the medical model conflict (51.05) obtained the highest and lowest scores, respectively. According to the multiple linear regression model analysis, the total mean score of midwives’ attitudes and beliefs toward physiological childbirth was significantly correlated with the level of education and interest in the profession (*P* < 0.05). Midwives with a master degree obtained higher scores (4.32) in terms of attitudes and beliefs toward physiologic childbirth, compared to those with an associate or bachelor degree. Also, there were 0.09 increases in the attitude and belief score of midwives per one score increase in their interest in the profession. It can be concluded that these two variables explained 16% of the variation in the scores of midwives’ attitudes and beliefs to physiological childbirth.

**Conclusion:**

The results of this study showed that midwives with higher levels of education and more interest in their profession had more positive attitudes and beliefs toward physiological childbirth. Therefore, it is necessary to motivate midwives to obtain higher levels of education and increase their interest in the profession to promote physiological childbirth.

## Background

Pregnancy and childbirth are the most important periods in a woman’s life [1]. Moreover, they are events which can have long-term physical, psychological, social, and emotional effects on individuals’ lives [2, 3]. Childbirth which is considered a common obstetric emergency is a natural and uncomplicated process [4]. However, with the advent of medical advances and increased safety in the twentieth century, the administration of medical interventions and use of the associated technology have become widespread in most of the childbirth cases [5].

Accordingly, over the past few decades, the medical model of childbirth has brought both a set of attitudes and beliefs. In this model, childbirth is regarded as a disease which requires medical intervention rather than a normal physiological process [6]. This attitude necessitates a situation where the onset of one intervention usually leads to additional interventions which consequently results in the implementation of a range of medical interferences [6].

These common medical interventions in physiological childbirth lead to an increase in unnecessary cesarean sections without any medical reasons which ultimately harm mothers and neonates [7, 8]. On the other hand, physiological childbirth is considered to be a safer mode of delivery. This mode of delivery is characterized by spontaneous onset and progression of labor without common interventions and medications [9]. In other words, it is a completely natural process requiring psychological support and accompaniment rather than management and intervention [10]. This kind of childbirth is commonly performed using different types of non-invasive pain relief techniques, such as relaxation, respiratory techniques, massage therapy, cold therapy, and heat therapy [11]. Benefits of physiological childbirth to mothers may include the increased levels of self-esteem, autonomy, intrapartum independence, physical and mental health, empowerment, and satisfaction, as well as reduced fear of delivery [12].

Moreover, the neonates benefit from physiological childbirth in terms of early breastfeeding [13] and the prevention of respiratory problems [14]. The implementation of physiological childbirth as a part of midwifery professional behavior is affected by several factors. According to the theory of planned behavior, the intention to perform a behavior is predicted by three factors, namely the attitude towards that behavior, subjective norms, and perceived behavioral control [15].

In a theory proposed by Ajzen and Fishbein (1980), attitudes and normative beliefs have been introduced as the predictive factors of behavioral intentions. These factors affect midwives’ care performance, behavior, and perinatal outcomes. Therefore, the adoption of more positive attitudes towards physiological childbirth by midwives leads to improved maternal and neonatal outcomes [16].

Beliefs are thoughts that one understands as truth or knowledge; they formulate and organize an attitude toward an entity. Ultimately, these beliefs form attitudes, behavioral intentions, and particular actions. Moreover, the attitudes have significant effects on shaping behaviors, generating motivation, satisfying the needs, and orienting the trends. They also interfere with intentions regarding the choice of performing a behavior [17].

Midwives as the first and most effective providers of prenatal and obstetric cares play significant roles in the process of delivery and labor, as well as perinatal outcomes. The promotion of midwives’ knowledge affects not only their attitude and beliefs toward physiological childbirth, but also their performance, and care quality, health behaviors, costs, and perinatal outcomes, thereby enhancing fertility in the community. Additionally, midwives’ positive attitudes toward physiologic childbirth make the training sessions effective, inform mothers, and make them more self-efficient in childbirth, breastfeeding, self-care, and neonatal care [16].

In a study conducted by Sauls (2007), nurses with more positive attitudes toward physiological childbirth provided mothers with greater support during labor [18]. Rosen-Carole et al. examined the knowledge and attitudes of service providers toward supporting breastfeeding mothers. Their results showed that positive knowledge and attitude toward breastfeeding had a positive effect on mothers to start breastfeeding and increase its duration [19].

In addition to other factors associated with attitudes and beliefs toward physiological childbirth, a study by Levine and Lowe revealed the association between nurses’ higher levels of education and more positive attitudes toward this practice [16]. Klein et al. (2011) compared the attitudes of two different age groups. They found that younger gynecologists and obstetricians supported the role of technology during physiological childbirth and paid less attention to the role of mothers in this regard. Moreover, the younger gynecologists had more positive attitudes toward intervention and technology utilization and paid less attention to the maternal role during childbirth [12]. However, midwives and doulas had more positive attitudes toward physiological childbirth owing to their long-term experience in this domain [20]. In the same line of research, Kiani et al. conducted a study to determine the attitude of midwives towards elective cesarean section and its related factors in 2014. The results showed that 24.26% of midwives had a positive attitude toward the elective cesarean section. Furthermore, a significant relationship was observed between the attitude of midwives towards elective cesarean section and choice of delivery type in hospitalized women (*P* = 0.001).

The results obtained from extensive surveys in different databases, however, revealed a dearth of research on the assessment of midwives’ attitudes and beliefs toward physiological childbirth (MABPC). Moreover, the unconfirmed validity or low reliability of the researcher-made questionnaires adopted in most of the previous studies or their qualitative design have limited the generalizability of their results. With this background, the present study was conducted to assess MABPC and its related factors in selected hospitals affiliated to three Universities of Medical Sciences in Tehran, Iran, during 2018.

## Methods

The present cross-sectional study included midwives working at 15 hospitals affiliated to three universities of medical sciences, namely Tehran, Shahid Beheshti, and Iran, located in Tehran (Additional file 1*:* Table S1). The sample size was estimated at 200 cases at a 95% confidence interval and 80% test power considering a Pearson correlation coefficient of 0.2 between MABPC and the predictive value. The sampling continued until the final sample was achieved from May 13 to July 16 in 2018. The inclusion criteria were: 1) a minimum of 6-month work experience in the labor and delivery wards and 2) participation in training courses on physiological childbirth. On the other hand, the participants with more than 10% skipped responses were excluded from the study.

The data were collected using a two-part demographic characteristic form and Nurses’ Attitudes and Beliefs Questionnaire-Revised (NABQ-R).

The 20-item demographic characteristic form covered such information as age (years), level of education (i.e., associate, bachelor, or master degree), work experience (year and month), labor-related work experiences (year and month), interest in profession (score range: 0-100), career satisfaction (score range: 0-100), shift work (i.e., either morning or night shift and rotational shift), and type of hospital (i.e., teaching hospital with and without the presence of an assistant and non-teaching hospital).

The NABQ-R was developed by Levine and Lowe (2015) and included 42 items with five domains, namely women’s experience of birth (10 items), women’s autonomy (12 items), medical model conflict (10 items), breech safety (3 items), and intervention influence (7 items). The respondents were asked to answer the questions based on a 4-point Likert scale ranging from strongly disagree (scored 1) to strongly agree (scored 4) with 12 reverse-coded items (i.e., items 3, 4, 5, 7, 8, 9, 13, 19, 20, 22, 23, and 24). Therefore, the instrument has minimum and maximum scores of 42 and 168, respectively. In this regard, a higher score is indicative of more positive MABPC [16]. Given the fact that this study examined the attitude and beliefs of midwives as a group with higher responsibility for providing care during pregnancy, labor, and birth in Iran [21], Levine and Lowe (2015) questionnaire was found to be a useful instrument to be utilized in this study [16].

The Persian version of MABQ-R (Midwives’ Attitudes and Beliefs Questionnaire-Revised) toward physiological childbirth was not available in Iran. Therefore, the designers’ permission was obtained, and the questionnaire was translated into Persian. Three translators translated the text into Persian; subsequently, two other bilingual interpreters translated the text from Persian to English. After comparing the translated text with the original one, the necessary revisions were made. To confirm the qualitative face validity of the questionnaire, 30 midwives were asked to check the items to revise the problematic or ambiguous ones. Also, 12 faculty members of Nursing and Midwifery School at Iran University of Medical Sciences, Tehran, assessed the questionnaire in terms of content validity which resulted in some revision in the questionnaire. Then the construct validity of the questionnaire was performed using confirmatory factor analysis (CFA), on 200 eligible midwives. The results of CFA showed that the Persian version of MABQ-R to physiological childbirth was well organized after the translation process.

Furthermore, time stability and internal consistency were used to determine reliability. In the test-retest method, 30 midwives completed the questionnaires twice with a time interval of 14 days. Furthermore, the intraclass correlation coefficient between the two measurements was obtained as 0.78. The internal consistency of the instrument was confirmed rendering a Cronbach’s alpha coefficient of 0.72. Therefore, the Persian version of MABQ-R was finally found to be valid and reliable instrument.

The study protocol was approved by the Ethics Committee of Iran University of Medical Sciences (IR.IUMS.FMD. 1396.9511373005). Furthermore, informed written consent was obtained from the participants, and the respondents were completely informed of the study purpose and procedures. Hence, they were assured of the confidentiality of their information. The questionnaires were completed by midwives.

Statistical analysis was performed in SPSS software (version 25). The quantitative variables were expressed as mean and standard deviation, and the categorical variables were presented as frequency and percentage.

After the confirmation of the normality of data, t-test, ANOVA, and Scheffe’s tests were utilized to analyze the relationship of midwives’ attitudes and beliefs with their demographic characteristics. All variables with a *p*-value of less than 0.05 were imported into multiple linear regression model with a backward strategy to determine the variations and estimate the effect of independent variables (i.e., demographic characteristics) on the dependent variable (i.e., MABPC). The assumptions of linear regression, such as the normalized residue ratio scale, homogeneity of residual variance, collinearity of outliers, and independence of the residues, were assessed before performing multivariate statistical analysis.

## Results

In total, 220 midwives were eligible to participate in this study; however, 20 participants were excluded from the research due to having more than 10% skipped responses. Finally, the study was conducted on 200 midwives. Table 1 summarizes the total scores of MABPC and its domains. The items had a score range of 0-100, and the domains of women’s experience of birth and medical model conflict obtained the highest (80.43) and lowest (51.5) scores, respectively. Table 2 illustrates the demographic characteristics of the participants and their relationship with MABPC.

**Table 1:** Scores of midwives’ attitudes and beliefs toward physiological childbirth and its domains (*n* = 200)

Midwives’ attitudes and beliefs and its domains	Mean	SD	Minimum	Maximum
Women’s experience of birth (10-40)	33.55	3.50	26	40
Women’s experience of birth out of 100	78.53	11.67	53.33	100
Women’s autonomy (12- 48)	34.76	3.76	28	46
Women’s autonomy out of 100	63.23	10.46	44.44	94.44
Medical Model Conflict (10-40)	25.31	3.23	16	34
Medical Model Conflict out of 100	51.5	10.77	20	80
Breech safety (3-12)	7.99	1.82	3	12
Breech safety out of 100	55.52	20.33	0	100
Intervention influence (7-28)	18.21	3.10	8	28
Intervention influence out of 100	53.40	14.78	4.76	100
Total attitude and belief score (42-168)	119.90	9.30	95	144

**Table 2:** Relationship of midwives’ demographic characteristics with their attitudes and beliefs toward physiological childbirth (*n* = 200)

Characteristic/variable	N (%)	*P*-value*
Age (years)		† 0.08
<30	54 (27.8)	
30 - 39	64 (33.0)	
≥40	76 (39.2)	
Level of education		‡ **< 0.001**
Associate and bachelor degree**s**	174 (87/0)	
Master degree	26 (13.0)	
Work experience (years)		† **0.01**
<5	53 (26.5)	
5 - 9.99	51 (25.5)	
10 - 14.99	27 (13.5)	
15 - 19.99	21 (10.5)	
≥20	48 (24.0)	
Work experience in labor (year)		† **0.02**
<5	77 (39.5)	
5 - 9.99	54 27.7)	
10 - 14.99	17 (8.7)	
15 - 19.99	20 (10.3)	
≥20	27 (13.8)	
Interest in the profession		† **0.01**
<50	34 (17.2)	
50 - 75	57 (28.8)	
≥76	107 (54.0)	
Career satisfaction		† 0.13
<50	114 (58.5)	
50 - 75	38 (19.5)	
≥76	43 (22.0)	
Type of hospital		† 0.86
Teaching with the presence of an assistant	145 (72.5)	
Teaching without the presence of an assistant	116 (58.0)	
Non-teaching	55 (27.5)	
Shift work		‡ **0.03**
Either morning or night shift	47 (32.3)	
Rotational	153 (67.7)	

*Significance level: *P* < 0.05

†One-way ANOVA test was used to determine the relationship of midwives’ attitudes and beliefs with their demographic characteristics

‡Independent sample t-test was used to determine the relationship of midwives’ attitudes and beliefs with their demographic characteristics

Bold entries are significant results and are related to statistical tests

According to the obtained results, MABPC was not significantly correlated with age, type of hospital, and career satisfaction. However, the results revealed that MABPC was significantly correlated with the level of education (*P* < 0.001), work experience (*P* = 0.01), work experience in labor (*P* = 0.02), interest in the profession (*P* = 0.01), and shift work (*P* = 0.03).

This study assessed the association between different variables and MABPC. Therefore, those variables with statistically significant associations with MABPC were imported into a multiple linear regression model with backward strategy. Among the associated factors, the level of education and interest in the profession remained in the model. According to the results, midwives with a master degree obtained higher scores (4.32) in terms of attitudes and beliefs toward physiologic childbirth, compared to those with associate or bachelor degrees. Also, the scores of midwives’ attitudes and beliefs increased to 0.09 by adding one score to the midwives’ interest in the profession. Consequently, based on the results of the study, 16% of the variation in the dependent variable (i.e., MABPC) was justified by the independent variables, namely the level of education and interest in the profession (Table 3).

**Table 3:** Effect of midwives’ demographic characteristics on their attitudes and beliefs toward physiological childbirth based on the results of multiple linear regression analysis

Independent variable		Unstandardized coefficients Beta	Standardized coefficients Beta	t	CI	*P*-value	R^2^
Level of education	Associate and bachelor degrees	Reference category					0.16
	Master degree	4.32	0.16	2.25	0.53, 8.11	**0.02**	
Work experience (year)		- 0.01	−0.01	−0.06	- 0.33, 0.31	0.95	
Work experience in labor (year)		0.11	0.10	0.79	- 0.17, 0.40	0.42	
Interest in the profession		0.09	0.22	3.25	0.03, 0.14	**< 0.001**	
Shift work	Either morning or night shift	Reference Category					
	Rotational	- 1.02	−0.04	- 0.61	- 4.30, 2.24	0.53	

Bold entries are significant results and are related to statistical tests

## Discussion

The present study is the first attempt addressing the investigation of Iranian MABPC. According to the results, the domains of women’s experience of birth and the medical model conflict obtained the highest and lowest scores, respectively. The present study is very similar to a study conducted by Levine and Lowe (2015) in terms of methodology and instrument utilization. Therefore, it is worthwhile to compare the results of these two studies to assess the differences in terms of MABPC in Canada and Iran. In the study performed by Levine and Lowe, the highest and lowest scores were assigned to women’s experience of birth (80.43) and intervention influence (43.95), respectively. This result is consistent with the findings of this study in which the women’s experience of birth obtained the highest score. However, the conflicting model of care and intervention influence in this study and the one performed by Levine and Lowe obtained the lowest scores, respectively. Based on our results and those obtained by Levine and Lowe, the most significant difference in terms of the conflicting model of care was observed in the mean scores of items 3, 4, and 22.

In this study, 76.9% of the midwives agreed with item 3 stating that “Midwives know better than mothers regarding the required pain relief method during labor” (mean score: 2.01). However, in the study conducted by Levine and Lowe (2015), most midwives paid more attention to mothers’ requests in choosing the pain relief method (mean score: 3.12). In the same line, Atghai et al. (2010) examined the attitude toward labor pain and the choice of delivery method in pregnant women referring to health centers in Kerman, Iran, using a researcher-made questionnaire. They found that 84% of women did not receive any training on pain relief methods during pregnancy [22]. According to the findings of the current study, it seems that mothers did not have the necessary knowledge about pain relief methods. Therefore, midwives did not consider mothers’ preferences in this regard and their opinions as trustworthy people were preferred to mothers’ choices.

Furthermore, the mean scores of item 4 (“Women would not choose physiological childbirth after cesarean section if they knew the consequences of uterine rupture”) were obtained as 2.11 and 3.03 in the present study and the study by Levin and Lowe, respectively. In the current study, 68.3% of the midwives agreed with the above-mentioned item. It seems that Iranian midwives recommend cesarean section to women with a previous cesarean. This is due to their concerns about the risk of uterine rupture and the following legal consequences for women.

In the study by Levin and Lowe (2015), only a few midwives agreed with this item which is not in line with our results [16]. In another study, almost half of care providers (48%) did not recommend physiological childbirth after previous cesarean section. The main reasons for avoiding or reducing the rate of physiological childbirth include the threatening nature of this delivery mode for maternal and fetal health (28%), as well as its associated medical and legal responsibilities (23%) [23].

In addition, the mean scores of item 22 (“The continuous fetal heart rate monitoring is preferred to intermittent auscultation during labor”) were estimated at 2.55 and 3.13 in the present study and the research by Levin and Lowe, respectively. Most Canadian midwives are opposed to electronic fetal heart rate monitoring. Given the fact that the onset of one intervention leads to successive interventions and this view is in contrast with the Canadian midwives’ positive attitudes toward physiological childbirth [16]. According to the results of the mentioned studies, the differences in midwives’ attitudes to electronic fetal monitoring could be associated with the level of the hospital, the influence of other service providers, shortage of midwives, overcrowding in the workplace, fear of taking responsibility, and legal accusations [24, 25].

Furthermore, McNiven et al. (2011) examined Canadian midwives’ attitudes toward physiological childbirth. In both studies, the midwives had positive attitudes toward reducing the rates of cesarean section and supporting the doulas (higher than 90%). Moreover, the midwives agreed with the implementation of intervention during normal labor progress by performing epidural anesthesia (88.4% in the study by McNiven vs. 66.2% in the present study). The results of a study carried out by McNiven showed midwives’ supports in terms of physiological childbirth (91%), delivery at home and out-of-hospital childbirth centers (99%), the role of women in labor, and lack of routine interventions (100%) [26].

Based on the results of this study, the midwives had positive attitudes toward birth planning, physiological childbirth benefits, reduced rates of cesarean section, epidural analgesia interference with labor progression, mother’s role in childbirth, and supporting the doulas. The midwives are considered to be an important part of the maternity care system. Accordingly, the identification of midwives’ responsibility to take care of low-risk mothers leads to higher levels of motivation among midwives to encourage physiological childbirth with no intervention. These observed differences between the results of the present study and those of other studies conducted in other countries resulted from midwives’ inappropriate working conditions, lack of support in terms of facility and performance [27], lack of implementation of policies to support physiological childbirth, fear of legal accusation [23], lack of mothers’ awareness about the benefits of physiological childbirth, and maternal willingness to the implementation of intervention, such as cesarean section [28].

About the factors associated with MABPC, there was no correlation between age and the mean of MABPC in this study and the research conducted by Levin and Lowe (2015). However, Vedam et al. (2010) found a significant correlation between age and American MABPC (*P* < 0.001) which can be due to the increased the mean of ages among participants (49 years).

In the present study, there was a significant relationship between the level of education and the mean of MABPC. Therefore, the midwives with higher levels of education (i.e., master degree) had more positive attitudes and beliefs toward physiological childbirth. In the study performed by Levin and Lowe (2015), there was a significant correlation between the level of education and the total mean score of midwives’ attitudes and beliefs (*P* < 0.001).

The results of this study showed no significant relationship between the mean of MABPC and the type of hospital. Similarly, in the study performed by Levin and Lowe (2015), there was no statistically significant relationship between the total mean score of midwives’ attitudes and beliefs and the level of the hospital. However, Liva et al. (2012) examined the factors associated with the adoption of different attitudes toward labor and delivery by Canadian nurses. They found that nurses’ attitudes to childbirth were influenced by the caregivers who were their companions at work and the level of hospital care. The MABPC was found to be affected by referring high-risk childbirths to third-level hospitals, access to interventions, the presence and supervision of other providers of services on labor.

In the present study, there was a significant relationship between the mean of MABPC and work experience. In this respect, the midwives with 20 or more years of work experience had more positive attitudes and beliefs toward g physiological childbirth. In the study conducted by Liva et al. (2012), a significant reverse correlation was reported between the work experience and nurses’ attitudes and beliefs toward childbirth in terms of electronic fetal monitoring (*P* < 0.001) and episiotomy (*P* < 0.01).

The findings of the present study also showed a significant relationship between the mean of MABPC and increased work experience in labor care. Furthermore, the results of a study by Stark and Miller (2009) demonstrated a significant relationship between increased work experience in labor care and the elimination of barriers to the use of hydrotherapy in labor (*P* = 0.01) [29].

In the present study, there was a significant relationship between the mean of MABPC and the interest in the profession (*P* = 0.01). In this regard, the midwives who obtained a score range of 76-100 in the interest in the profession had more positive attitudes and beliefs toward physiological childbirth. It should be noted that there were no other studies to compare the results in this domain.

According to the obtained results in the present study, there was no significant relationship between the mean of MABPC and career satisfaction score (*P* = 0.13). There were no other studies to compare the results in this domain.

Moreover, in the present study, there was a significant relationship between the mean of MABPC and shift work. The midwives with either morning or night shift had more positive attitudes and beliefs toward physiological childbirth. However, the results of a study conducted by Levin and Lowe (2015) showed no significant relationship between MABPC and shift work. In Iran, the experienced midwives with more work experience in constant shifts had more positive attitudes toward physiological childbirth. On the other hand, the rotational shifts are assigned to the other midwives with fewer years of work experience.

The present study was carried out in selected hospitals affiliated to Tehran University of Medical Sciences, Tehran, Iran. It was assumed that due to the availability of facilities in these hospitals, the use of technology and interventions were more prevalent. However, the findings revealed midwives’ positive attitudes to physiological childbirth and negative attitudes to the utilization of common interventions and technology, especially among those with higher levels of education and more interest in the profession. Midwives are regarded as important agents in the health care system during pregnancy and childbirth that play remarkable roles in the implementation and promotion of physiological childbirth. Therefore, the presence of midwives who are interested in their careers is worthwhile. Also, it is necessary to motivate midwives to obtain higher levels of education and acquire the required skills toward physiological childbirth.

Although this study paved the way for addressing Iranian MABPC, it suffers from some limitations. In this study, the MABPC were assessed in public hospitals. Therefore, future studies are recommended to evaluate MABPC in private hospitals. It is recommended that further studies compare midwives and specialists’ attitudes and beliefs toward physiological childbirth and its associated factors. Furthermore, further studies are suggested to compare this issue between trained and untrained midwives.

## Conclusion

Midwives are regarded as an important part of the health system for providing women with services during labor, delivery, and postpartum. Moreover, they play a remarkable role in the implementation and promotion of physiological childbirth. Therefore, midwives must gain higher levels of education and increase their level of interest in the profession to promote physiological childbirth.

## Additional file

**Additional file 1: Table S1.** Frequency distribution of midwives in selected hospitals in Tehran, Iran, during 2018. (DOCX 21 kb)

## Data Availability

No additional data available due to confidentiality restrictions.

## Abbreviations

CFA: Confirmatory Factor Analysis
MABPC: Midwives’ Attitudes and Beliefs toward Physiological Childbirth
MABQ-R: Midwives’ Attitudes and Beliefs Questionnaire-Revised
NABQ-R: Nurse Attitudes and Beliefs Questionnaire-Revised
