# Supplementary material for: Iranian midwives’ attitudes and beliefs toward physiological childbirth: a cross-sectional study
Source: BMC Pregnancy Childbirth. 2019 Oct 12;19:352. doi: 10.1186/s12884-019-2509-y (PMC6790054; doi:10.1186/s12884-019-2509-y)
Supplement: Supplementary file 1 — Additional file 1: Table S1. Frequency distribution of midwives in selected hospitals in Tehran, Iran, during 2018. (DOCX 21 kb) [file 12884_2019_2509_MOESM1_ESM.docx]

**Additional file 1: Table S1. Frequency distribution of midwives in selected hospitals in Tehran, Iran, during 2018**

| **Hospitals** | **Frequency** | **Percentage** |
| --- | --- | --- |
| Shahid Akbar Abadi | 31 | 15.5 |
| Mahdiyeh | 25 | 12.5 |
| Ziaian | 18 | 9.0 |
| Baharlu | 15 | 7.5 |
| Fatemeh Al-Zahra | 15 | 7.5 |
| Firouzabadi | 13 | 6.5 |
| Lolagar | 13 | 6.5 |
| Imam Hossein | 13 | 6.5 |
| Taleqani | 12 | 6.0 |
| Emam Sajjad | 11 | 5.5 |
| Arash | 10 | 5.0 |
| Shohadaye Tajrish | 8 | 4.0 |
| Yas | 6 | 3/0 |
| Firoozgar | 5 | 2.5 |
| Rasoul Akram | 5 | 2.5 |
| Total | 200 | 100 |
